# Supplementary material for: Comprehensive Annotation and Functional Exploration of MicroRNAs in Lettuce
Source: Front Plant Sci. 2021 Dec 24;12:781836. doi: 10.3389/fpls.2021.781836 (PMC8739914; doi:10.3389/fpls.2021.781836)
Supplement: Supplementary file 3 [file Data_Sheet_9.ZIP › GSM2230753.plot/Lsa-miR172c_Lsat_1_v5_gn_3_126700.1_453_TPlot.pdf]

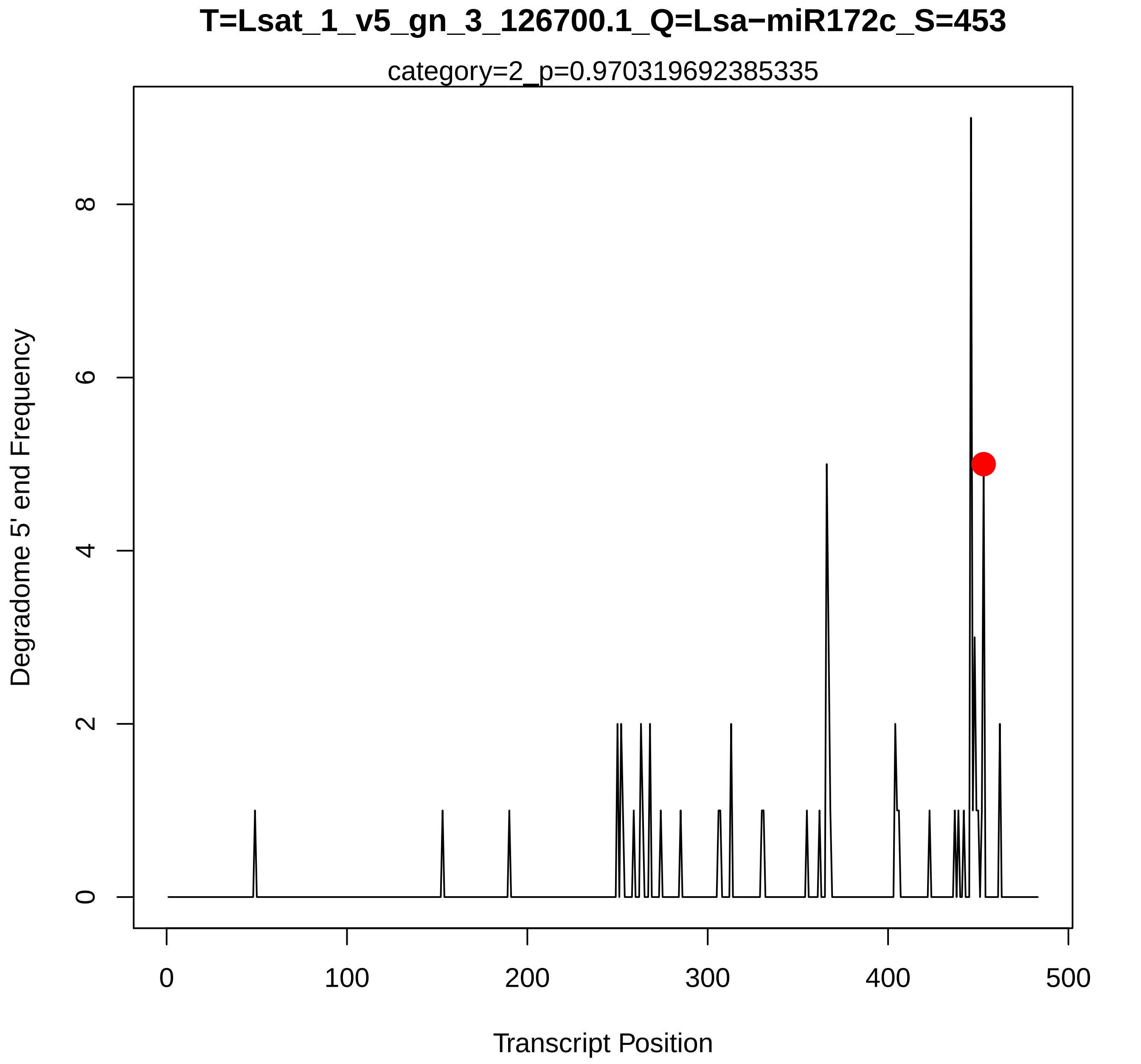
T=Lsat_1_v5_gn_3_126700.1_Q=Lsa−miR172c_S=453
category=2_p=0.970319692385335
Degradome 5' end Frequency
0
2
4
6
8
0
100
200
300
400
500
Transcript Position
